# Boosting Cuproptosis in Breast Cancer Therapy via Photodynamic Treatment With a New Liposome

**DOI:** 10.1096/fba.2025-00280

**Published:** 2026-02-17

**Authors:** Jie Yu, Ning Sun, Limei You, Jialing Liu, Mengna Niu, Jiacheng Shi, Weixin Chen, Futong Li, Shengbao Wang, Jiaqi Liu

**Affiliations:** ^1^ Department of Pharmacology Mudanjiang Medical University Mudanjiang Heilongjiang China; ^2^ Department of Pharmacy Mudanjiang Medical University Mudanjiang Heilongjiang China

**Keywords:** breast cancer, Cuproptosis, liposomes, photodynamic therapy, synergistic treatment

## Abstract

Breast cancer (BC) is one of the most common cancers in women around the world, and utilizing a combined approach is a crucial strategy. Induction of cuproptosis in tumor cells is a novel antitumor approach, though its standalone efficacy remains unclear. In this study, we prepared a novel liposome loaded with the photosensitizer indocyanine Green (ICG) and the cuproptosis inducer elesclomol‐Cu (ES‐Cu) to examine the synergistic effects of photodynamic‐cuproptosis treatment on BC. The cuproptosis inducer ES‐Cu and the photosensitizer ICG were encapsulated in nanoliposomes with a membrane hydration approach and then validated in vitro and in vivo. JC‐1, MDA, GSH, and other cuproptosis‐related indicators were used to confirm the ability of PDT to enhance ES‐Cu‐induced cuproptosis in MCF‐7 breast cancer cells. For confirming the cytotoxic impact of PDT in conjunction with the cuproptosis inducer, tests for CCK‐8 and cell death staining were performed. The drugs were administered to animals via tail vein injection to observe their tumor inhibition effects in vivo. Their safety was assessed by monitoring changes in body weight. The average particle size of liposomes loaded with ES‐Cu and ICG was 208.3 ± 1.07 nm, exhibiting a consistent nanospherical morphology. ICG produced cytotoxic reactive oxygen species (ROS) that enhanced ES‐Cu‐induced cell cupping under NIR laser irradiation. The therapeutic effect of the synergistic treatment combining PDT and cuproptosis was validated in both in vitro and in vivo experiments. This investigation proved that PDT markedly augments the ES‐Cu‐induced cuproptosis in breast cancer cells, demonstrating a synergistic therapeutic effect. This synergistic effect presents a novel therapy approach for BC with substantial practical application potential.

## 1 Introduction

As one of the most frequent malignant tumors among women around the world, the high incidence along with mortality rates of breast cancer (BC) pose a major challenge to public health [1]. Conventional therapies, for example, radiotherapy, chemotherapy, and surgery, although effective to some extent, have many side effects and treatment tolerance issues [2].

Cuproptosis is a recently identified kind of cell death connected to the copper ion accumulation in cells [3]. A recent study has found that induction of cell death using copper ions is a potential cancer treatment strategy [4]. Cancer cells normally display higher oxidative stress and metabolic activity, making them more sensitive to copper ion toxicity [5]. Extracellular Cu^2+^ is bound and transported to intracellular compartments by the copper ionophore Elesclomol [6]. FDX1 prevents the formation of Fe‐S cluster proteins by reducing Cu^2+^ to Cu^+^ [7]. As an upstream regulator of the fatty acylation of proteins, FDX1/LIAS can regulate the fatty acylation of mitochondrial enzymes like DLAT [8]. Cu^+^ binds to acylated DLAT in the TCA cycle, inducing the accumulation of fatty acylated proteins [9]. Proteotoxic stress and eventual cell death are the results of these abnormal processes taken together [10]. Leveraging this property, therapeutic drugs can be developed to selectively accumulate copper ions in cancer cells, thereby improving treatment specificity and effectiveness [11]. However, copper ions lack specificity for tumor cells and may impact healthy cells as well, resulting in harmful side effects and systemic toxicity [12].

Photodynamic therapy (PDT) has exhibited great promise as a new noninvasive therapeutic approach in recent years [13]. PDT utilizes light activation to irradiate the tumor site locally, minimizing harm to normal tissues and increasing therapy safety and specificity [14]. However, PDT's limitations in treating breast cancer include poor selectivity of photosensitizers, limited light penetration depth, and challenges with metabolism and excretion [15]. Using PDT in conjunction with other therapies allows for the comprehensive utilization of their respective advantages, overcoming the shortcomings of single therapy and improving overall breast cancer treatment outcomes [16]. Combining cuproptosis with photodynamic therapy may represent a novel cancer treatment strategy. Cuproptosis induces copper ion accumulation in cells, triggering cytotoxicity and cell death, while PDT kills tumor cells by producing reactive oxygen species through photosensitizers activated by specific wavelengths of laser [17]. The combination of copper ion‐induced oxidative stress and PDT‐generated ROS causes a synergistic effect, potentially resulting in stronger cytotoxicity and improved therapeutic outcomes.

Liposomes are nanoscale drug carriers widely used in tumor treatment due to their unique bilayer membrane structure and excellent biocompatibility [18]. Liposome drug delivery technology can improve drug stability and bioavailability, enhance targeting at tumor sites, and reduce treatment side effects [19]. This study developed a method to deliver ICG and ES‐Cu using liposomes, aiming to achieve photodynamic sensitization and cuproptosis in breast cancer treatment. ICG is a near infrared photosensitizer with excellent optical properties and biocompatibility, while ES‐Cu induces cell death by chelating copper ions [20, 21]. Encapsulating these two drugs in liposomes is expected to improve their stability and targeting in tumor tissues, improve the effectiveness of the treatment, and minimize the side effects on normal tissues. This article will explore the design, mechanism, and potential of this liposomal drug delivery system for breast cancer treatment. To the best of our knowledge, using ICG and ES‐Cu‐loaded liposomes for combined copper‐photodynamic therapy of HCC cells is a new method that has never been reported in prior research.

## 2 Materials and Methods

### 2.1 Materials

1, 2‐distearoyl‐sn‐glycero‐3‐phosphoethanolamine‐N‐[methoxy (poly (ethylene glycol))‐2000] (DSPE‐ PEG2000), cholesterol (Chol), phosphatidyl ethanolamine (PE), and lecithin (PC) were provided by Avanti Polar Lipids (Alabaster, AL). Indocyanine green (ICG) and elesclomol (ES) were supplied by Sigma‐Aldrich (Shanghai, China). JC‐1 kit and AM/PI kit were supplied by Beyotime Biotechnology. CCK‐8 was acquired from BD Pharmingen.

### 2.2 Preparation of ICG@ES‐Cu Lip

To fabricate the ICG & ES‐Cu‐loaded nanodrugs, PE, PC, DSPE‐PEG2000, and Chol with the molar ratio of 20:50:5:25 (a total of 30 mg) and 1 mg of ES‐Cu were dissolved into 2.0 mL of chloroform (CHCl3), then 0.2 mL of pH 7.4 PBS with ICG (5 mg) was incorporated into the solution while it was being sonicated (20 kHz, 30% power level, 5‐s on, 2‐s off) in an ice‐cold bath for 2 min. Next, 20 mL of pH 7.4 PBS solution was mixed with the resulting emulsion solution under the same sonication condition for 2 min. Finally, a centrifugal filter device (MWCO: 100 kDa) was employed to wash the solution three times with new PBS after a rotary evaporator removed the CHCl_3_. After filtration via a 0.20 μm syringe filter, the solution containing ICG & ES‐Cu co‐loaded nanodrugs (ICG@ES‐Cu Lip) was obtained. ICG loaded nanodrugs were abbreviated to ICG@Lip.

### 2.3 Characterization

A transmission electron microscope (TEM; Hitachi, Japan) was exploited to investigate the form and distribution of ICG@ES‐Cu Lip. A dynamic light scattering (DLS) analyzer (ZEN3600; Malvern Instruments) can be applied to investigate its zeta potential and particle size distribution.

### 2.4 Cell Culture

MCF‐7 cells were acquired from the Chinese Academy of Sciences Cell Bank in Shanghai, China. They were cultivated in DMEM media with 10% FBS and maintained at 37°C with 5% CO_2_ in a humid incubator.

### 2.5 Assessment of the In Vitro Anticancer Activity of Various NPs

MCF‐7 cells were cultivated for 24 h after being inoculated into 96‐well plates at 1 × 10^4^ cells/well. After incubating for an hour, the media was taken out and the cells were treated with several doses of ICG@ES‐Cu Lip (1, 5, 10, 20, 40, 80, 120, 160, 200 μg/mL). Each well was washed with PBS three times. Three sets of cell wells were divided: ICG@ES‐Cu Lip only, ICG@Lip+NIR, and ICG@ES‐Cu Lip+NIR. The parameters of the laser were as follows: wavelength (808 nm), output intensity (1.0 W/cm^2^), and irradiation period (5 min/well). Cell viability was calculated with CCK‐8 assay.

### 2.6 Cell Live/Dead Cell Labeling

Hepa1‐6 cells were divided into five groups: Control group; ICG@Lip group; ICG@ES‐Cu Lip group; ICG@Lip + NIR group and ICG@ES‐Cu Lip+NIR group. The laser has the same power as previously stated. After that, the cells were stained using calcein‐AM/PI for 20 min. A fluorescence microscope was exploited to view the cellular fluorescence.

### 2.7 Measurement of the Potential of the Mitochondrial Membrane

The mitochondrial potential was determined by applying the JC‐1 (Beyotime, C2005) detection kit. The experimental grouping is the same as the live cell/dead cell labeling section. After being exposed to an ice‐cold PBS solution, the tagged cells were examined via a fluorescence microscope.

### 2.8 Analysis of GSH

Following different treatments (PBS, ICG@Lip; ICG@ES‐Cu Lip; ICG@Lip+ NIR; ICG@ES‐Cu Lip+ NIR), the cells were trypsinized after three PBS washes. Gather the cells and centrifuge twice for 5 min at 4°C and 1000 rpm. Resuspend each milligram in protein removal solution M (3 μL) based on the weight of the cell pellet. Following vortexing, freeze and thaw it twice quickly in a water bath at 37°C and liquid nitrogen and then place it at 4°C. Centrifuge at 10,000 rpm for 5 min. The amount of GSH present in the sample is ascertained by analyzing the supernatant. To initiate the reaction, add NADPH to the system after preparing the proper quantity of detection working solution along with the supernatant sample as directed, mixing them, and letting them sit at room temperature (RT) for 5 min. Detect the absorbance of TNB at 412 nm after an hour, then compute the level of GSH as directed.

### 2.9 MDA Assay

Gather the protein in MCF‐7 through various treatments (PBS, ICG@Lip; ICG@ES‐Cu Lip; ICG@Lip + NIR; ICG@ES‐Cu Lip + NIR), and 40 min after lysis following centrifugation, 200 μL was transferred to a 96‐well plate, the supernatant was combined with the TBA detection working solution, heated for 15 min in a boiling water bath, allowed to cool to RT, and the absorbance was determined at 532 nm. The content of MDA was then measured by comparing the standard curve.

### 2.10 ROS Detection

After seeding MCF‐7 cells in 6‐well plates and cultivating them overnight for the removal of the media, the cells were treated for an additional four hours with ICG@ES‐Cu Lip, ICG@Lip, PBS, ICG@ES‐Cu Lip + NIR, and ICG@Lip + NIR. After that, DCFH‐DA was given to the cells, and they were kept out of the light for 30 min at 37°C. The cells were exposed to laser radiation for 30 s (1.0 W/cm^2^) following the media removal. They were then rinsed twice with DMEM, given 1 mL of cold PBS, and rapidly examined under a fluorescence microscope. Cells from a 6‐well plate are digested for flow cytometry, and the procedure is carried out as mentioned earlier.

### 2.11 Antitumor Effect In Vivo

Due to the convenience of the subcutaneous model for real‐time monitoring of tumor growth and drug distribution, it was employed in this study for preliminary efficacy assessment. The liposomes were administered via tail vein injection at a dose of 5 mg/kg (based on ICG), once every 4 days, for a total of 3 administrations. The tumor site was irradiated with an 808 nm laser at a power density of 1.0 W/cm^2^, once daily for 5 min per session. The first irradiation was performed 24 h after drug administration. Subcutaneous MCF‐7‐luc xenografts (up to approximately 100 cm^3^ in length) from BALB/c nude mice were separated into four groups following three separate treatments for in vivo tumor treatment. Body weight and tumor volumes were detected once every four days during the trial. The formula tumor volume = length × 1/2 × width^2^ was used to get the tumor volume at the same time, IVIS (AniView SE, Guangzhou Biolight Biotechnology Co. Ltd) was used to calculate bioluminescence signals in vivo.

### 2.12 Statistical Analysis

Each experiment was conducted three times. The biological data are displayed as percentages and are calculated as mean ± SD of three replicates. Student's *t*‐test in GraphPad Prism 8.0 was used to compare all sample values to the control (untreated cells) value, which represented 100% of all sample values. For multiple group comparisons, one‐way analysis of variance (ANOVA) followed by Tukey's post hoc test was used. To ascertain whether the control and sample values were extremely significant, very significant, or significant, *p* values of 0.001, 0.01, and 0.05 were employed, respectively.

## 3 Results

### 3.1 Synthesis and Characterization of ICG@ES‐Cu Lip

**FIGURE 1:**
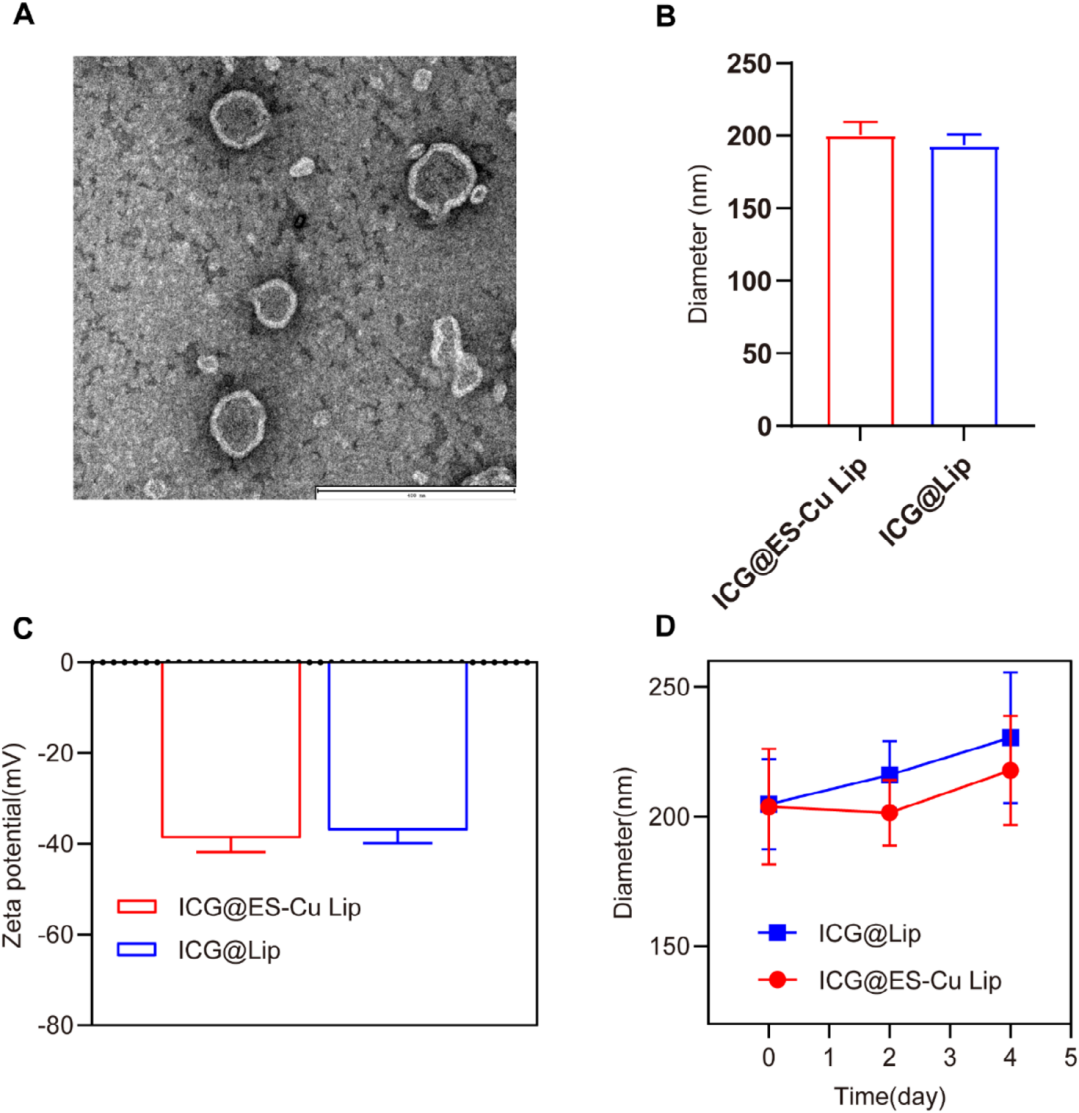
Characterization of ICG@ES‐Cu Lip. (A) TEM images displayed the quasi‐spherical morphology of ICG@ES‐Cu Lip, with scale bar of 400 nm; (B, C) DLS results presented the average zeta potential along with diameter size of ICG@ES‐Cu Lip. (D) Diameter size changes in ICG@ES‐Cu Lip placed in DMEM culture medium.

Figure 1A shows the morphology of ICG@ES‐Cu Lip obtained using transmission electron microscopy. The ICG@ES‐Cu Lip morphology is uniform and consistent, with a mean particle size and zeta potential of 208.3 ± 1.07 nm (Figure 1B) and −38.1 ± 1.01 mV (Figure 1C), respectively. In addition, we investigated the stability of ICG@ES‐Cu Lip in DMEM culture medium. As shown in Figure 1D, when ICG@ES‐Cu Lip was placed in DMEM culture medium for 4 days, its particle size did not change significantly.

### 3.2 ICG@ES‐Cu Lip Can Induce Cuproptosis in MCF‐7 BC Cells

**FIGURE 2:**
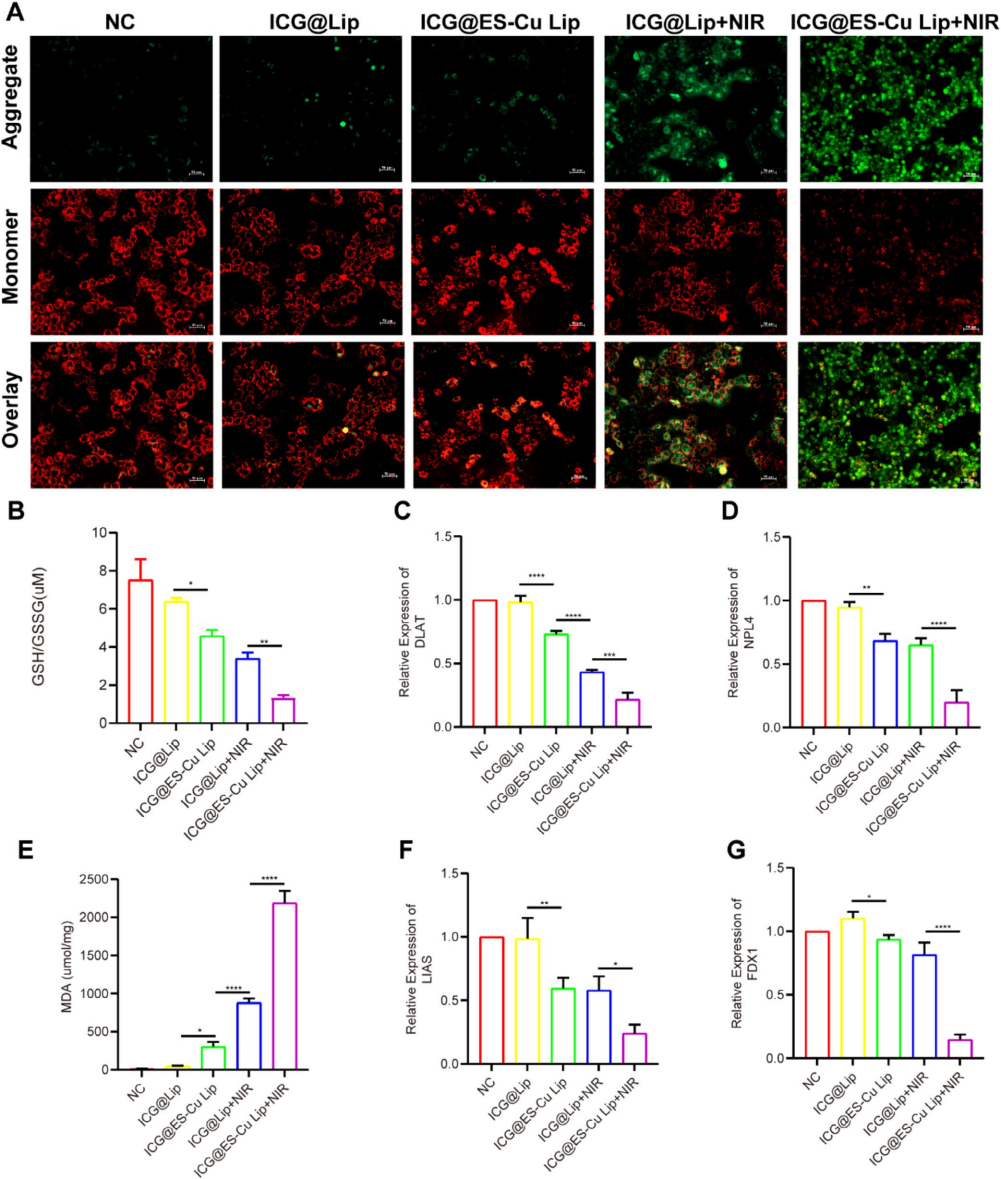
Functional analysis of ICG@ES‐Cu Lip and MMP in cuproptosis. (A) Fluorescence microscopy images of JC‐1 aggregates (red channel) and monomers (green channel) in HCC cell mitochondria under various treatments; (B, E) DTNB assay for the MDA detection and GSH level in HCC cells under various treatments; (C, D, F, G) qRT‐PCR analysis of mRNA expressions in DLAT, NLP4, LIAS and FDX1.

The cuproptosis process induces mitochondrial dysfunction, resulting in a reduction in mitochondrial membrane potential (MMP). As a fluorescent probe that examines MMP, JC‐1 displays variations in MMP by color change. The fluorescence of JC‐1 is red when the mitochondrial membrane potential is high, and green when the membrane potential is low. Therefore, JC‐1 staining can effectively detect this process. After incubation of cells with various treatments, fluctuations in MMP were evaluated through JC‐1 staining. Figure 2A revealed that in contrast to the other treatment groups, when ICG@ES‐Cu Lip was used in combination with near‐infrared laser (NIR), a marked rise in green fluorescence and an evident reduction in red fluorescence were noted, suggesting a clear rise in mitochondrial membrane potential. In addition, GSH levels are commonly applied to assess the ferroptosis intensity. As shown in Figure 2B, the GSH level in the ICG@ES‐Cu Lip + NIR group was significantly reduced. Meanwhile, MDA is the final product of late‐stage fat oxidation. Figure 2E presents that the expression levels of MDA increased continuously during the treatment; There was a negative relationship between GSH and them. Finally, we explored the enhancing effect of PDT on cuproptosis by detecting the expression of key genes in the cuproptosis process. The results show that in the ICG@ES‐Cu Lip + NIR group, the mRNA expression levels of key cuproptosis genes DLAT (Figure 2C), NLP4 (Figure 2D), LIAS (Figure 2F), and FDX1 (Figure 2G) significantly decreased, consistent with the process by which cuproptosis occurs. The above results indicate that PDT can enhance cuproptosis in MCF‐7 cells.

### 3.3 Measurement of Intracellular ROS in SDT

**FIGURE 3:**
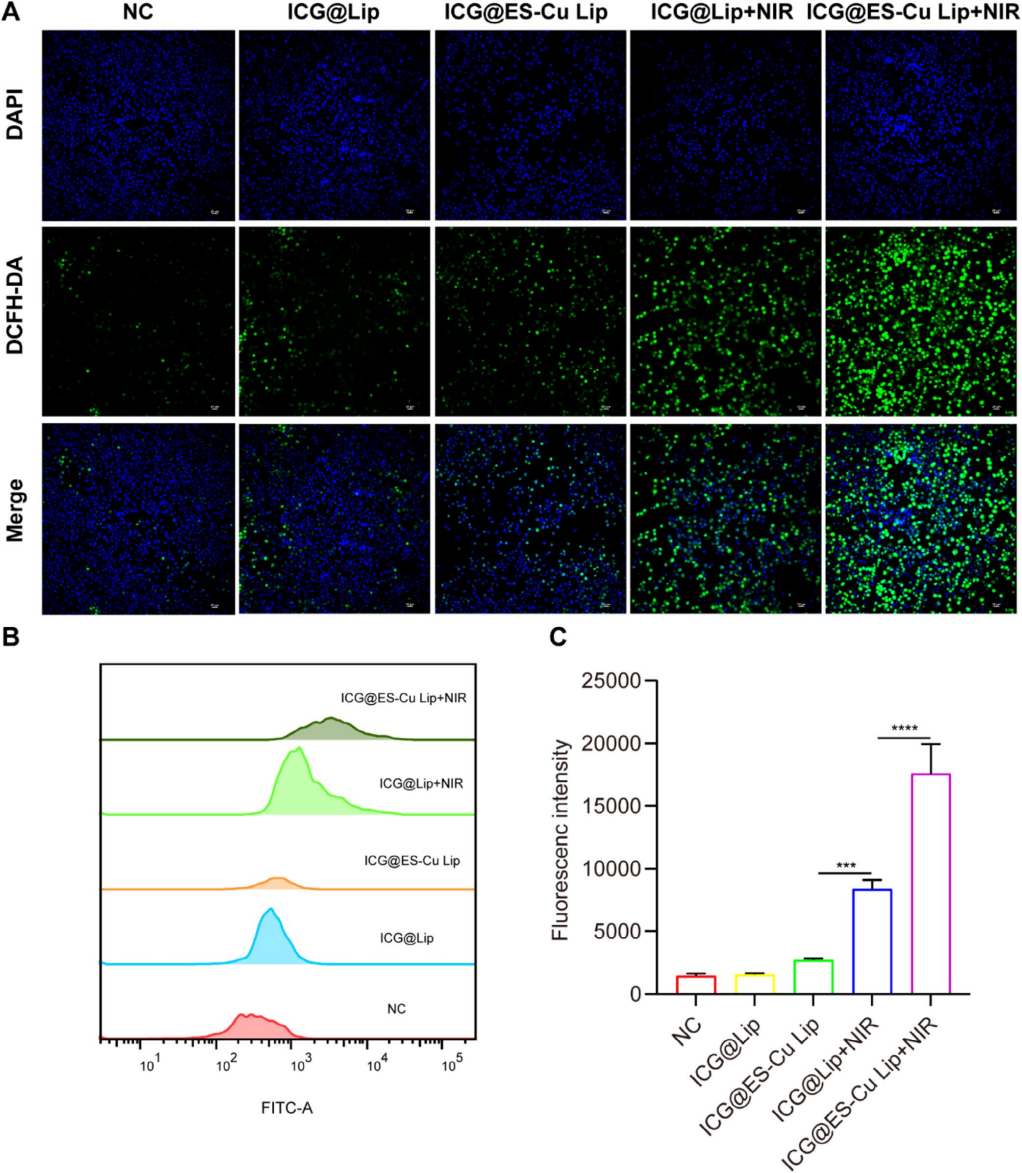
ROS analysis. (A) Fluorescence microscopy images of DCFH‐DA assay for the generation of intracellular ROS after receiving various treatments; (B, C) Flow cytometry analysis of the generation of intracellular ROS in the same experiments described previously.

In the present work, we utilized a DCFH‐DA fluorescent probe to assay intracellular ROS generation by fluorescence microscopy and quantitatively analyzed the ROS levels by flow cytometry to verify the oxidative stress effect of liposome‐delivered ICG and ES‐Cu combined treatment on breast cancer cells. Under a fluorescence microscope, it is evident that when treating MCF‐7 cells with ICG@ES‐Cu Lip + NIR, they show the most fluorescence (Figure 3A). Besides, flow cytometry also displayed that MCF‐7 cells in the ICG@ES‐Cu Lip + NIR group had the highest fluorescence intensity (Figure 3B,C), which was in agreement with the IF results. The above findings exhibit that PDT can enhance cuproptosis.

### 3.4 Cytotoxicity of Therapeutic Effect In Vitro

**FIGURE 4:**
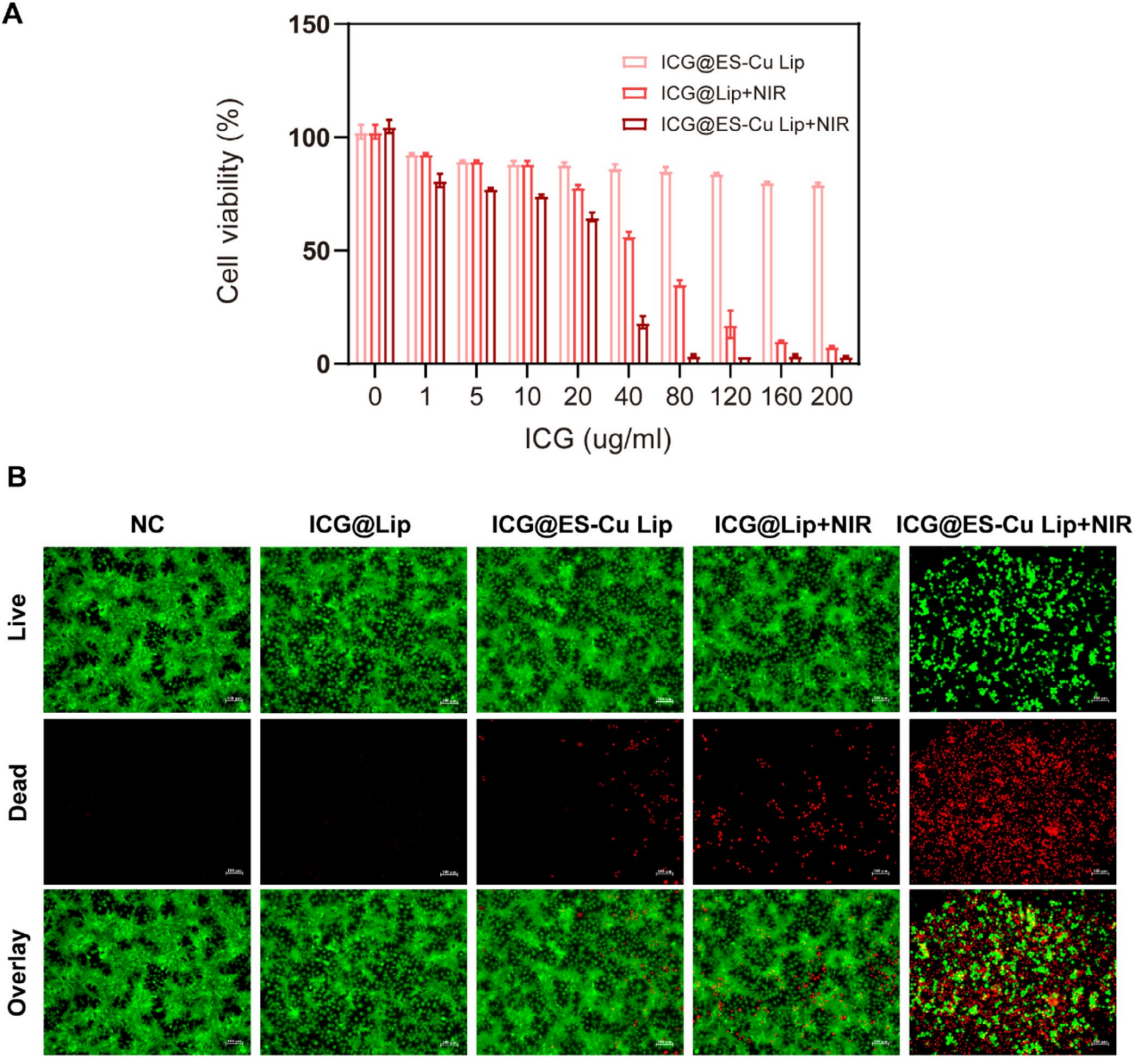
In vitro efficacy analysis. (A) Cytotoxicity analysis of differential treatment with various concentrations of ICG@ES‐Cu Lip or ICG@Lip. Data are expressed as mean ± SD (*n* = 3); (B) Representative fluorescence images (scale bar = 100 μm) of cells that were stained with propidium iodide (red, dead cells) and calcein‐AM (green, live cells) in separate treatments.

When used in combination, the copper ion‐induced oxidative stress and ROS produced by PDT have a synergistic effect, potentially leading to stronger cytotoxicity and thus improving the therapeutic effect. Therefore, the cytotoxicity of various NPs in the presence and absence of PDT treatment was assessed. Figure 4A indicates that even though the concentration of NPs raised, the survival of MCF‐7 cells did not change significantly when using ICG@ES‐Cu Lip alone. However, under the same power of NIR irradiation, there was a remarkable difference in cytotoxicity between ICG@ES‐Cu Lip and ICG@Lip, and the ICG@ES‐Cu Lip + NIR group had the best antitumor effect. Tests utilizing live/dead cell staining were then carried out to examine the antitumor action in more detail. Figure 3B presents that MCF‐7 cells with ICG@ES‐Cu Lip + NIR treatment showed a large amount of red fluorescence, indicating that it caused a large amount of tumor cell apoptosis, which was in line with the findings of the CCK‐8 assay. The above results indicate that PDT can evidently increase the antitumor effect induced by cuproptosis.

### 3.5 In Vivo Antitumor Effect

**FIGURE 5:**
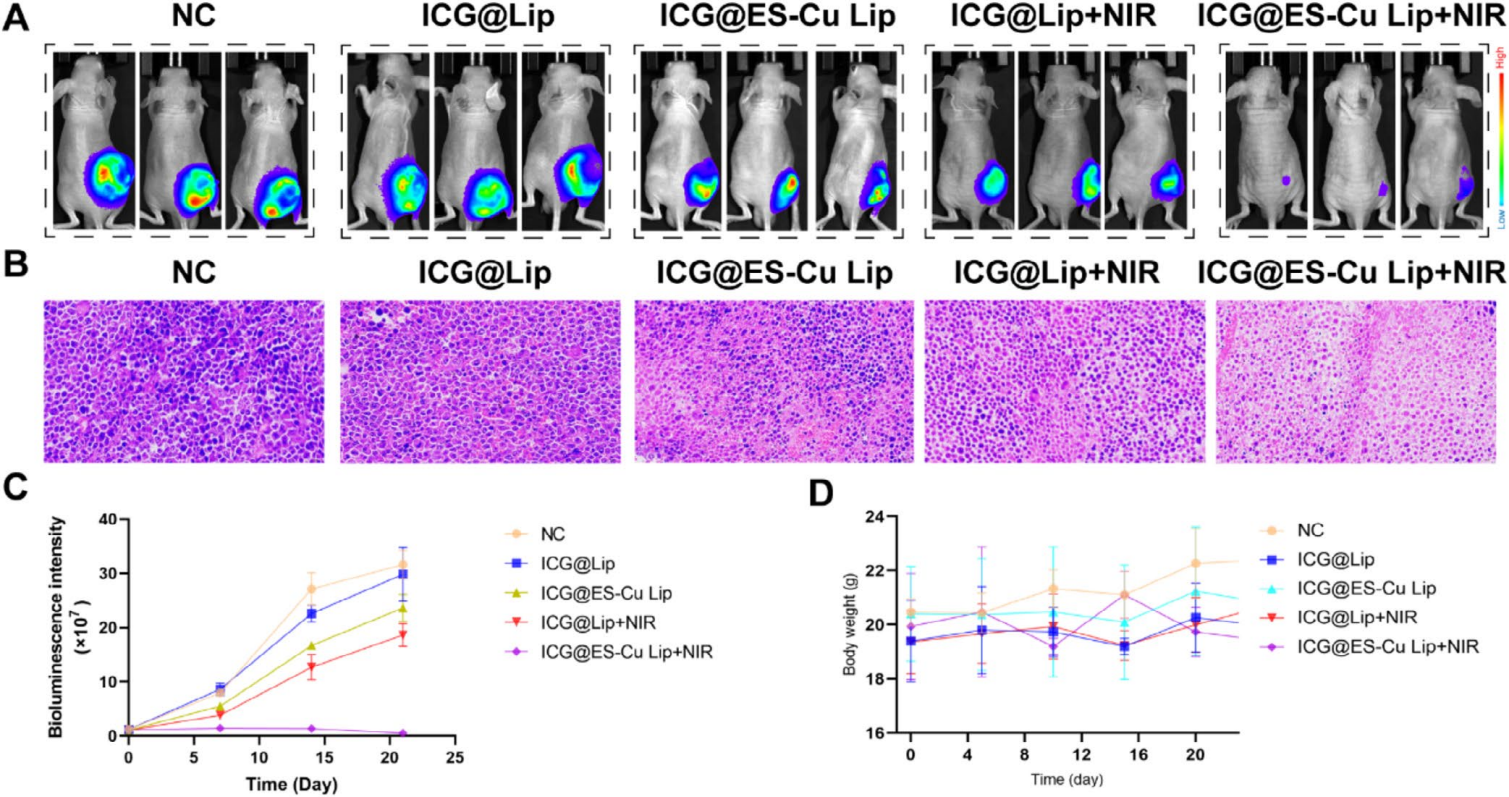
Efficacy of in vivo synergistic treatment. (A) Xenograft breast cancer in various groups of tumor‐bearing mice on Day 21 of the follow‐up period (*n* = 3). (B) H&E Staining of xenograft breast cancer. (C) In vivo bioluminescence images quantified signal intensity of the xenograft breast cancer in mice during treatment (*n* = 3). (D) The weights of tumor‐bearing mice following each treatment are illustrated in the figure.

**FIGURE 6:**
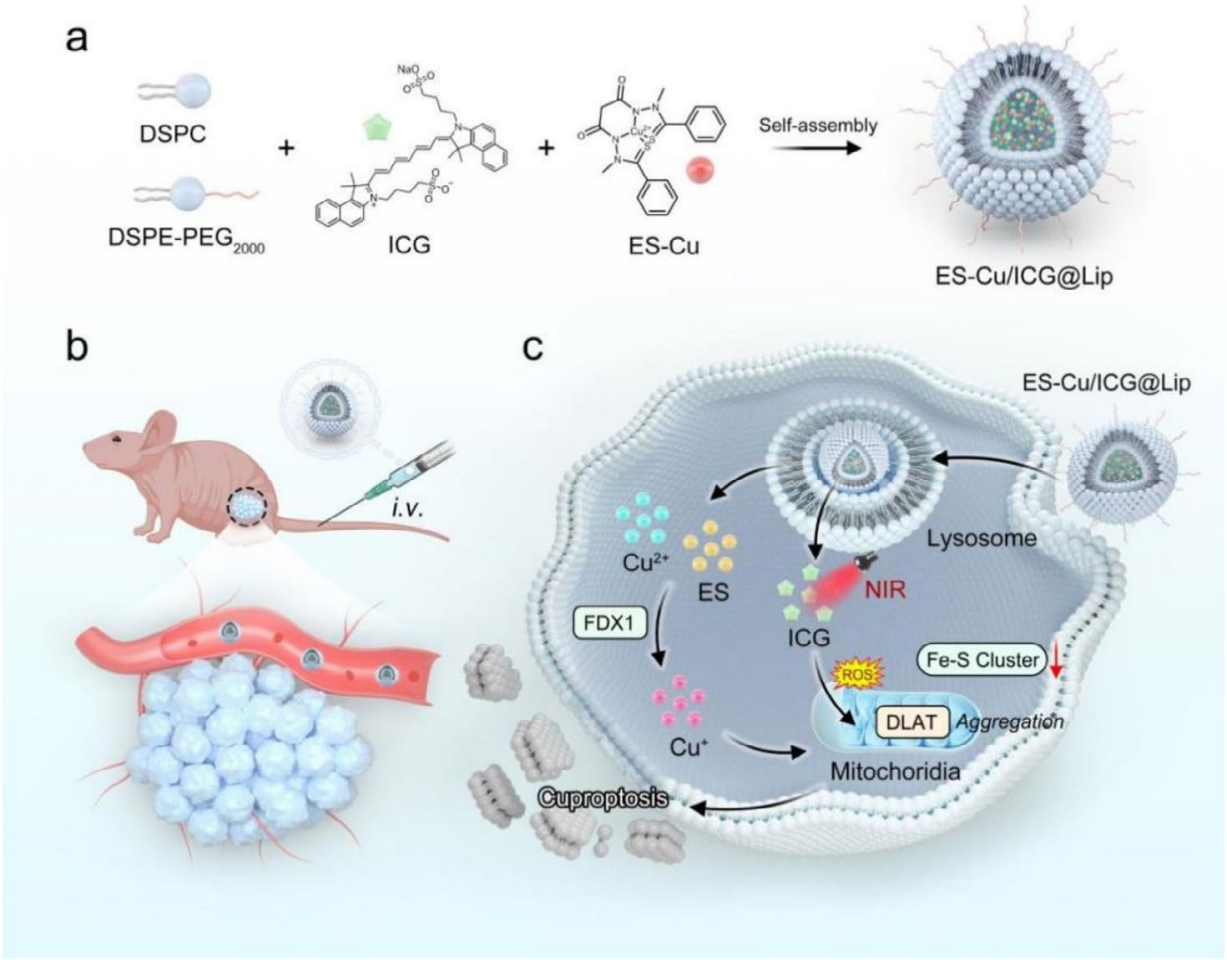
Schematic mechanism of enhanced cuproptosis in breast cancer via novel liposomal photodynamic therapy. (a) Self‐assembly process of ES‐Cu/ICG@Lip liposome: DSPC, DSPE‐PEG2000, ICG, and ES‐Cu form a multifunctional liposome through self‐assembly. (b) In vivo delivery of the liposome: intravenous injection (i.v.) into a breast cancer mouse model for tumor targeting. (c) The intracellular mechanism of action of liposomes. The liposome is internalized by cells and releases Cu^2+^, ES, and ICG in lysosomes. Near‐infrared light (NIR) excites ICG to generate reactive oxygen species, synergizing with Cu^+^ to disrupt mitochondrial metabolism. This induces tumor cell cuproptosis by inhibiting FDX1, destroying Fe‐S clusters, and blocking the DLAT‐mediated lipoylation pathway.

By measuring variations in tumor size, this study assessed the anticancer effects of different treatment regimens. As displayed in Figure 5A,C, the treatment effects are ranked from lowest to highest as follows: NC group = ICG@Lip < ICG@ES‐Cu Lip < ICG@Lip + NIR < ICG@ES‐Cu Lip+NIR. We observed ICG@ES‐Cu Lip treatment had minimal effect on mitigating tumor growth, whereas the ICG@ES‐Cu Lip+NIR treatment group exhibited significantly stronger tumor inhibitory effects. For further demonstrating the antitumor effect of ICG@ES‐Cu Lip therapy, we performed HE staining on tumor tissues (Figure 5B). The findings suggested that the tumor tissue sections of the ICG@ES‐Cu Lip+NIR group showed significant tissue structural changes. Massive tumor cells were necrotic, the cell nuclei disappeared, and the cytoplasm showed obvious degeneration and disintegration, suggesting that the combined treatment induced by PDT and cuproptosis triggered a strong cell killing effect. Additionally, owing to maintaining biosafety is essential to general health and well‐being, in vivo biosafety tests were carried out to evaluate parameters such as body weight. The aforementioned indices did not significantly alter across all treatment groups, as exhibited in Figure 5D. In summary, these findings indicated that the PDT markedly augments the ES‐Cu‐induced cuproptosis in breast cancer cells, demonstrating a synergistic therapeutic effect (Figure 6).

## 4 Discussion

Combination therapy has displayed remarkable benefits in the treatment of BC in recent years [22]. Combination therapy can address the limitations of single‐drug therapy and mitigate drug resistance [23]. Cuproptosis, as a novel anticancer treatment strategy, induces apoptosis and necrosis by chelating copper ions, demonstrating significant antitumor effects across various cancer types [24]. Nevertheless, the therapeutic effect of copper ions alone is limited by their release kinetics and biological activity within the body [25]. Photodynamic therapy (PDT), a noninvasive local treatment method, kills tumor cells by activating photosensitizers to produce reactive oxygen species and has garnered significant attention in cancer treatment [26]. However, due to the hypoxic and high‐GSH state of the breast cancer tumor microenvironment (TME), single PDT is ineffective. Encouragingly, we have found that the substantial ROS generated by PDT could potentially amplify the cellular oxidative stress induced by cuproptosis agents, thereby bolstering the antitumor efficacy [27]. Therefore, in this study, we prepared a liposomal nanodrug, ICG@ES‐Cu Lip, to co‐deliver the cuproptosis inducer ES‐Cu and ICG. ICG@ES‐Cu Lip was employed to assess the potential of combined PDT and copper ion‐induced cell death (cuproptosis) for BC treatment in both in vivo and in vitro models. DSPE‐PEG2000, PC, PE, and Chol were chosen as raw materials for this study. ICG and ES‐Cu were encapsulated in their respective hydrophilic cavities and hydrophobic layers to form ICG@ES‐Cu liposomes, which had a particle size of 208.3 ± 1.07 nm (Figure 1B). TEM images (Figure 1A) verified the spherical morphology and size distribution range of the liposomes.

We employed multiple approaches to evaluate the capacity of PDT to enhance cellular cuproptosis. First, during cuproptosis, the accumulation of copper ions damages mitochondria, causing a reduction in mitochondrial membrane potential (Δψm). The JC‐1 reagent can indirectly reflect mitochondrial membrane damage and cell death induced by intracellular copper ions by measuring changes in mitochondrial membrane potential [28]. The results indicate that ICG@ES‐Cu Lip combined with NIR significantly reduces mitochondrial membrane potential and enhances the mitochondrial damage caused by ES‐Cu (Figure 2A). Additionally, malondialdehyde (MDA) is a well‐established marker of lipid peroxidation, which occurs during oxidative stress [29]. Because copper‐induced cell death (cuproptosis) involves ROS production and consequent oxidative damage [30], measuring MDA levels provides indirect evidence of cuproptosis (Figure 2E). By neutralizing ROS, glutathione (GSH), an essential antioxidant, shields cells from oxidative damage [31]. GSH depletion is a marker of oxidative stress and serves to confirm the occurrence of cuproptosis [32]. During cuproptosis, excessive ROS production overwhelms antioxidant defenses, resulting in GSH depletion (Figure 2B). The study results demonstrate that ICG@ES‐Cu Lip combined with NIR produces the highest MDA levels and significantly reduces GSH levels, indicating that PDT can significantly enhance ES‐Cu‐induced cuproptosis. Finally, we measured the mRNA expression levels of cuproptosis‐related markers (Figure 2C,D,F,G). FDX1 is a small iron–sulfur protein that is involved in the decrease of Cu^2+^ to Cu^+^ [7]. Furthermore, FDX1 is a crucial regulator of cuproptosis and an upstream regulator of the TCA cycle protein lipoylation pathway [33]. Dihydrolipoamide S‐acetyltransferase (DLAT) is a part of the pyruvate dehydrogenase (PDH) complex [34]. The TCA cycle integration of copper and lipoylated proteins leads to DLAT oligomerization [7]. In mitochondria, lipoic acid synthase (LIAS) produces the potent antioxidant α‐Lipoic acid (LA) and encodes elements of the lipoic acid pathway [35].

We also explored the potential mechanism by which PDT enhances cuproptosis. Studies have shown that PDT induces tumor cell damage and death by generating ROS [36]. Elesclomol induces oxidative stress and disrupts normal cellular metabolic processes [37]. The experimental results indicate that the combined therapy of ES‐Cu and PDT dramatically raises the level of ROS in tumor cells (Figure 3A,B). The ROS levels were significantly higher in the combined treatment (ICG@ES‐Cu Lip + NIR) than in PDT alone (ICG@Lip + NIR) or in ES‐Cu (ICG@ES‐Cu Lip). Therefore, we propose that PDT further amplifies the intracellular oxidative stress response triggered by ES‐Cu by increasing ROS production, thereby enhancing copper‐induced cell death.

In the in vitro toxicity assessment, experimental findings of live‐death staining and CCK‐8 revealed that ICG@Lip + NIR showed higher toxicity than PDT or ES‐Cu treatment alone at the same ICG concentration (Figure 4A,B). The outcomes of in vivo antitumor experiments demonstrated that tumor growth in the ICG@Lip + NIR treatment group was only slightly slowed, indicating that the effect of PDT alone in inhibiting tumor growth is limited. ICG@ES‐Cu Lip also cannot significantly inhibit tumor proliferation, indicating that the antitumor effect of inducing cuproptosis in tumor cells is also limited. The combined therapy of cuproptosis and PDT has a considerable synergistic impact in reducing tumor development, as seen by the much higher tumor inhibitory effect of the ICG@ES‐Cu Lip + NIR treatment group in contrast to the other groups (Figure 5A–C). In addition, through monitoring the body weight of the tumor‐bearing mice, no drastic weight changes were observed in the experimental animals, indicating that ICG@ES‐Cu Lip + NIR therapy has good biosafety (Figure 5D).

## 5 Conclusion

In summary, this study explored the double‐hit effect on breast cancer cells by combining the cuproptosis strategy with PDT, using liposomes to co‐deliver ICG and ES‐Cu. Experimental results showed that PDT significantly enhanced the cuproptosis effect induced by ES‐Cu in tumor cells by increasing the production of ROS. Specifically, the combination treatment significantly increased intracellular ROS levels and copper ion accumulation, resulting in higher tumor cell mortality. This synergistic effect presents a new therapy approach for breast cancer with substantial practical application potential.

## Author Contributions

Jiaqi Liu contributed to the study conception and design. Jie Yu contributed to the data analysis and the writing of the first draft of the manuscript. Ning Sun and Limei You contributed to the material preparation work. Jialing Liu, Mengna Niu, and Jiacheng Shi contributed to performing the biological experiments. Weixin Chen, Futong Li, and Shengbao Wang contributed to performing the cell and animal experiments.

## Funding

This study was supported by The Doctoral Scientific Research Foundation of Mudanjiang Medical University (No. 2021‐MYBSKY‐053, 2022‐MYBSKY‐003) and Scientific Research Projects for Basic Scientific Research in Heilongjiang Provincial Universities (no. 2023‐KYYWF‐0912).

## Ethics Statement

The study protocol of animal trials conformed to the ethical guidelines of the 1975 Declaration of Helsinki and was approved by the Animal Care and Ethics Committee of Mudanjiang Medical University (20230103–6).

## Conflicts of Interest

The authors declare no conflicts of interest.

## Data Availability

Stored in repository.
